# Participants’ views of ultra-low dose combination therapy for high blood pressure: a mixed-methods study from the QUARTET trial

**DOI:** 10.1038/s41371-024-00915-4

**Published:** 2024-05-14

**Authors:** Joshua G. Kovoor, Clara K. Chow, Abdul Salam, Ruth Webster, Louise Shiel, Mark R. Nelson, Jacquita S. Affandi, Peter Hay, Michael Burke, Gemma A. Figtree, Tim Usherwood, Christopher M. Reid, Markus P. Schlaich, Anthony Rodgers, Emily R. Atkins

**Affiliations:** 1 Westmead Applied Research Centre, Faculty of Medicine and Health, University of Sydney, Sydney, NSW Australia; 2 Ballarat Base Hospital, Ballarat, VIC Australia; 3 Westmead Hospital, Sydney, NSW Australia; 4 The George Institute for Global Health, UNSW, Sydney, NSW Australia; 5 The George Institute for Global Health, Hyderabad, Telangana India; 6 Prasanna School of Public Health, Manipal Academy of Higher Education, Manipal, India; 7 School of Population Health, UNSW, Sydney, NSW Australia; 8 School of Public Health and Preventive Medicine, Monash University, Prahran, VIC Australia; 9 Menzies Institute for Medical Research, University of Tasmania, Hobart, TAS Australia; 10 Curtin School of Population Health, Curtin University, Perth, WA Australia; 11 Castle Hill Medical Centre, Sydney, NSW Australia; 12 Kildare Road Medical Centre, Sydney, NSW Australia; 13 Kolling Institute, Royal North Shore Hospital, Sydney, NSW Australia; 14 Charles Perkins Centre, University of Sydney, Sydney, NSW Australia; 15 Dobney Hypertension Centre, Medical School – Royal Perth Hospital Unit and RPH Research Foundation, The University of Western Australia, Perth, WA Australia

**Keywords:** Risk factors, Hypertension, Medical research

## Abstract

Single-pill combination therapy containing four quarter-dose medications for high blood pressure improves BP control compared to monotherapy, however patient-reported acceptance of the quadpill as a treatment strategy remains undescribed. We collected within-trial feedback and interviewed participants from the quadruple ultra-low-dose treatment for hypertension (QUARTET) trial to characterise patient attitudes to this intervention. All trial participants were asked about ease and preference for the quadpill and provided an opportunity to give further comments on the trial at 12 weeks (trial primary endpoint) and 52 weeks extended follow-up. Separately, we used purposive and quota sampling for the semi-structured telephone interviews, with the resultant verbatim transcripts analysed using an inductive thematic analysis approach. Themes were re-evaluated after each successive interview, and at suspected data saturation, an additional interview conducted for confirmation. At 12 weeks follow-up, 502 of 591 (85%) participants responded to acceptability questions, and 359 of 417 (86%) responded at week 52. Most reported the trial capsule easy or very easy to take. From eight sites, 16 participants were interviewed between 5 August 2020 and 19 November 2020. All described a positive experience, preferred once-daily morning dosing and found routine facilitated adherence. Participants valued individual responsibility for adherence, and involvement of the general practitioner in blood-pressure management. Most reported capsule size did not deter adherence but desired a smaller capsule. Participants described a preference for minimising number and dosage of medications, reduced capsule size, and once-daily morning dosing. These findings suggest a preference for single-pill combination therapy for blood pressure lowering.

## Introduction

Raised blood pressure affects over 1.13 billion people worldwide [1], and is a leading preventable contributor to global morbidity and mortality [2]. Blood-pressure lowering therapies reduce the risk of fatal and non-fatal cardiovascular disease [3]. Multiple blood pressure lowering medications are needed to appropriately manage high blood pressure. This is recognised and recommended in multiple international guidelines [4, 5]. However, this can come with increased pill-burden, known to affect adherence. Medication non-adherence presents a significant obstacle to achieving optimal blood pressure control and cardiovascular event prevention.

Combining fixed doses of multiple active pharmaceutical ingredients within a single tablet or capsule is an established therapeutic method in many conditions. Single-pill combinations (SPCs) seek to reduce pill burden and improve adherence to constituent drugs [6]. Low and ultra-low dose SPCs of three or more blood-pressure lowering agents provide clinical improvements in the efficacy and tolerability compared to current therapy [7–10]. The quadruple ultra-low-dose treatment for hypertension (QUARTET) trial demonstrated initial treatment with a quadpill of four blood pressure lowering drugs at quarter dose (irbesartan 37.5 mg, amlodipine 1.25 mg, indapamide 0.625 mg, bisoprolol 2.5 mg) had greater blood-pressure lowering efficacy than starting with monotherapy, an effect sustained one year after randomisation [11, 12].

Broadly, there appears to be acceptability of cardiovascular SPCs in patients and caregivers, citing simplicity, and improved adherence, across a variety of healthcare settings but mostly in the context of reducing pill burden while maintaining existing combination therapy [13–15]. However, there have been no studies of patient acceptability of the quadpill, and very few of low-dose combination blood pressure lowering SPCs in the context of no previous treatment, or previous treatment with monotherapy. We conducted a mixed methods evaluation to characterise patient acceptability and attitudes of the quadpill, and identify factors that may influence implementation for initial treatment of high blood pressure. We also sought feedback on the trial methods as part of ongoing quality improvement of our trial processes.

## Methods

The QUARTET trial was a phase 3 double-blind randomised controlled trial comparing initial treatment of high blood pressure with an ultra-low-dose combination of four blood pressure lowering drugs at quarter dose (irbesartan 37.5 mg, amlodipine 1.25 mg, indapamide 0.625 mg, bisoprolol 2.5 mg) to a standard dose monotherapy (irbesartan 150 mg). Participants had high blood pressure and were previously untreated or on monotherapy. Depending on randomised allocation, the study capsules either contained the four quarter doses, or a standard dose plus three placebos. Capsules were provided at no cost to participants during the study visit or mailed. The trial was registered with Australian New Zealand Clinical Trials Registry (ACTRN12616001144404) and the protocol and primary results have been published [11, 12]. Five hundred and ninety-one participants were randomised, 417 continued into extended follow-up to 12 months. The quadpill was more effective than monotherapy at 12 weeks (trial primary endpoint) with systolic blood pressure 6.9 mmHg lower in the quadpill group than monotherapy, and this effect was sustained out to 12 months extended follow-up [11].

**Fig. 1:**
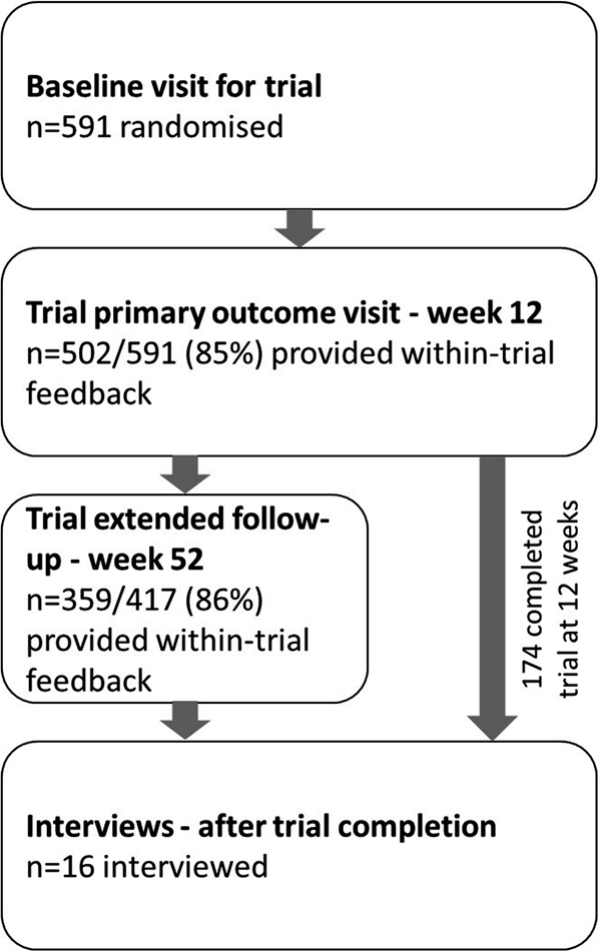
Study participant flow diagram. Five hundred and ninety-one participants were randomised at baseline. At week 12, 502 (85%) provided within-trial feedback during the primary outcome visit. Of the 417 who went into extended follow-up, 359 (86%) of participants provided within-trial feedback. One hundred and seventy-four participants completed the trial at 12 weeks. Sixteen participants completed interviews after trial completion.

These analyses were planned before the trial commenced. The interview guide was prepared alongside the trial protocol and within-trial feedback questions included in the case report forms [10]. We obtained informed consent from all research participants. Ethical approval was obtained from the Western Sydney Local Health Network Human Research Ethics Committee (HREC/15/WMEAD/422). We collected within-trial feedback during the study, and conducted interviews after the study had finished, but before the analysis of main results had completed (Fig. 1). Analysis of both was planned for after the trial had finished.

### Research team and reflexivity

The within-trial feedback questions, and the interview guide were developed by ERA and AS with review from other study investigators involved in clinical trials of low-dose combination blood pressure lowering therapy (CC, AR, RW). Another investigator (JGK), not known to the participants, conducted interviews over the telephone. He was a medical student at the time.

### Within-trial feedback

At weeks 12 and 52, during the study visit, all trial participants were asked to indicate on a five-point scale (i) how easy they found it to take the study capsule and (ii) how likely they would be to request the quadpill if available to be prescribed. Free-text comments about the treatment were collected. This was captured by site staff in the database during the trial, with the data released for analysis at completion of the trial, following the primary analysis.

Baseline characteristics of the respondents to within-trial feedback and interviewees were reported as means and standard deviation for continuous variables, and number with percentage for categorical variables. Feedback responses are reported as number with percentage, with cells of only one response aggregated with the next level. Analyses were conducted using SAS Enterprise Guide 7.1 (SAS Institute Inc., Cary, NC, USA). Participants’ free-text responses were coded into themes by one investigator (ERA).

### Interviews

Prior to study commencement, informed by the study objectives and previous studies [14, 16], we developed an interview guide containing 27 open questions and permitting follow-on ‘probe’ questions (Supplementary Appendix I).

All QUARTET trial participants (both quadpill and monotherapy arms) were eligible for inclusion at trial completion. As QUARTET was a double-blind trial, the participants did not know their treatment allocation, and they were not unblinded before the interviews. The interviews were conducted independently to the within-trial feedback. A combination of purposive and quota sampling strategies was used to recruit at least one participant from all QUARTET trial sites enroling at least 10 participants, and ensuring women were included [17]. Potential participants were selected and approached by their QUARTET site coordinators to gauge interest to participate. Those who were interested were then approached by the interviewer (JGK) via telephone or email, informed verbal consent was obtained, and interview scheduled at the participant’s convenience.

Each interview began by confirming consent, including permission to begin audio recording and transcription of conversations. Interviews were audio-recorded using QuickTime (Apple, Cupertino, CA, United States) and transcribed on Word (Microsoft Corporation, Redmond, WA, United States). The interviewer took notes during the interview to assist personal conceptualisation and transcribed verbatim all verbal data using the audio recording after each interview’s conclusion. All transcriptions and notes were de-identified to ensure interviewee confidentiality, and each interviewee numbered chronologically for reporting. Transcripts were not returned to participants. Emergent themes were re-evaluated after each successive interview, and interviewees were recruited until no new themes emerged and data saturation was reached [18]. At the point of suspected data saturation, an additional interview was conducted for confirmation.

The study was underpinned by a thematic analysis framework outlined by Braun and Clarke [19], carried out within an essentialist paradigm. We incorporated the primary and secondary validity criteria proposed by Whittemore et al. [20]. and Sekhon et al’s theoretical framework of acceptability [21]. Themes were identified by the interviewer using an inductive (data-driven) approach focused at a semantic level [19]. Using NVivo version 12 (QSR International, Melbourne, VIC, Australia), transcripts were coded line by line by the interviewer to develop a coding tree comprising major and minor themes. As themes emerged during analysis of each successive data element, the interviewer repeatedly revised the conceptual framework. The verbatim transcripts were checked against the themes by one investigator (ERA).Participant feedback was not sought on the study findings. The standards for reporting qualitative research checklist is included in the appendix [22].

## Results

### Baseline characteristics

Baseline characteristics (pre-randomisation in the trial) of interviewees and within-trial feedback respondents are presented in Table 1. Participants did not know their treatment allocation. There was an even split of participants randomised to intervention and control groups.

**Table 1:** Baseline characteristics of respondents and interviewees prior to randomisation.

	Within-trial feedback respondents *n* = 502	Interviewees *n* = 16
Randomised allocation, *n* (%)		
Quadpill	246 (49%)	8 (50%)
Monotherapy	256 (51%)	8 (50%)
Age, mean (SD) years	58 (11.4)	56 (12.3)
Female, *n* (%)	201 (40%)	6 (38%)
Ethnicity, *n* (%)		
White	411 (82%)	15 (94%)
Asian	59 (12%)	1 (6%)
Other	32 (6%)	–
Country of birth, *n* (%)		
Australia	323 (64%)	13 (81%)
Other	178 (36%)	3 (19%)
Employment status, *n* (%)		
Full-time	249 (50%)	9 (56%)
Part-time	65 (13%)	2 (13%)
Retired	147 (29%)	5 (31%)
Other	41 (8%)	–
Baseline Office blood pressure, mean (SD) mmHg		
Systolic	152 (15.8)	146 (14.6)
Diastolic	89 (10.8)	91 (10.0)
Completed 12 months follow-up	411 (82%)	14 (88%)

### Within-trial feedback

At week 12, 502 (85%) participants responded to one or more acceptability questions, and 359 (86%) responded at week 52. The majority reported the trial capsule easy or very easy to take, with similar proportions by treatment allocation (Table 2). There were small differences in responses by age group (<65 years, ≥65 years), time since hypertension diagnosis (<1 year, ≥1 year), and baseline polypharmacy (<5 medications, ≥5 medications) but in all groups the majority reported the capsule was very easy to take and they were very likely to request it from their usual doctor if it was available (Supplementary Table 1). Most participants had no comments about treatment, 11% (week 12) and 13% (week 52) made positive comments such as, “no side effects”, “working well”, “happy (with) controlled BP”. Three (1%) at week 12 and six (2%) at week 52 commented negative experiences (e.g. “Disappointing”). Nine (2%; week 12) and five (1%; week 52) had neutral comments, such as, “felt no difference” and “no issues.”

**Table 2:** Response to feedback questions on trial medications.

	Week 12 *n* = 502		Week 52 *n* = 359	
	Quadpill *n* = 246	Monotherapy *n* = 256	Quadpill *n* = 178	Monotherapy *n* = 181
During the trial, how easy did the participant find it to take the trial medications				
Very easy	174 (71%)	196 (77%)	131 (74%)	144 (80%)
Easy	61 (25%)	50 (20%)	36 (20%)	31 (17%)
Average, difficult or very difficult	11 (4%)	10 (4%)	11 (6%)	6 (3%)
If the LDQT is available to be prescribed by participant’s usual doctor, how likely would the participant be to request it?				
Very likely	147 (60%)	140 (55%)	118 (66%)	92 (51%)
Likely	71 (29%)	84 (33%)	47 (26%)	65 (36%)
Average	20 (8%)	20 (8%)	9 (5%)	14 (8%)
Unlikely or very unlikely	7 (3%)	11 (4%)	4 (2%)	9 (5%)

### Interviews

We completed interviews with 16 QUARTET trial participants between 5 August 2020 and 19 November 2020. No further themes emerged after 15 interviews, one more participant was interviewed for confirmation of thematic saturation [18]. No repeat interviews were conducted. Participants were recruited from eight of the ten QUARTET trial sites across Australia. All potential participants approached agreed to participate and none withdrew. Interview duration ranged from 12 to 48 min (median: 17.5, IQR: 13.8–22.3). Average time from trial completion to interview was 266 days (range: 27–542 days). Fourteen interviewees had completed 12 month extended follow-up, two had completed the study at 12 weeks.

### Themes identified

Across the free-text responses and interviews we identified six themes characterising acceptability of SPC treatment to people with high blood pressure (Table 3): minimising medication, perceived effects, value of daily routine, significance of individual responsibility, importance of the primary care physician, size of the capsule. A seventh theme emerged of participants’ experience in the QUARTET trial.

**Table 3:** Themes characterising patient acceptability of single-pill combination blood pressure lowering therapy.

Theme	Important quotes
Minimising medication	“I don’t like taking medication at all … what appealed to me was giv[ing] a quarter dose and not overmedicating.” (interview 1) “It was very simple to take just one tablet a day … for old people you don’t want to be taking 25 tablets a day if you can have the same effect with one … I think a pinch of several different medications suits better than a bucketful of one.” (interview 2)
Perceived effects of the intervention	“[My biggest surprise was] how quickly my blood pressure stabilised.” (interview 10) “The results that I was getting with the medication were fantastic, I had absolutely no side effects at all.” (interview 13)
Value of daily routine	“I’ve just established a habit because of it … it was just having the pill every day and that was no dramas.” (interview 11) “Very easy because it just fit straight into my routine, I had to do nothing special about it, no problems there.” (interview 13)
Individual responsibility	“I live by myself so look after myself.” (interview 8) “There’s only my wife and I here, and we’re responsible for our own medications.” (interview 9)
Importance of the primary care physician	“My [primary care physician] is quite keen on blood pressure … management, and he wasn’t happy with my previous medication.” (interview 2) “My [primary care physician] is quite open to changes if I’m happy with it.” (interview 9)
Size of capsule	“[It] was a fairly large capsule compared to the tablets that I’m used to taking that was the only thing, I presume that if it was a marketable product at the end of things that it would be a smaller package.” (interview 9) “The [capsule] was a little bit bigger than what I used to take … [but] tablet size doesn’t particularly worry me.” (interview 14)
Positive experience in the QUARTET trial	“It was a thousand per cent better in the trial setting than outside of because the [trial staff were] more concerned, and they showed more empathy, and showed actual willingness to see me do well too. It wasn’t just about the trial; it was also about the best outcome for myself.” (interview 6) “I felt the care was very comprehensive … range of tests, heaps and heaps of questions, all very thorough. I didn’t get asked any when I went to see my local healthcare.” (interview 12)

### Theme 1: Minimising medication

Most interviewees wanted to minimise their medication. Minimising medication was noted as either reducing pill burden (*n* = 6) or avoiding high dosages (*n* = 3). One respondent who was newly diagnosed at the beginning of the trial said, “I don’t like taking medication…I take minimal medications…What appealed to me was it was looking to give a quarter dose and not over medicate” (interview 1). They “would have preferred to monitor [their] blood pressure and go off medication all together” instead of transitioning to another medication at the end of the trial. This view may be due to multiple medication changes after the trial “Because of this pandemic, the first blood pressure medication my doctor prescribed is not available, and they’d now given me another type”(interview 1). For participants taking one BP-lowering medication prior to the QUARTET trial, convenience was reported as “it was simply just changing one tablet for a different tablet” (interview 16). Amongst the 16 interviewees, eight (50%) reported taking multiple medications in addition to their BP-lowering treatment, mostly for cardiovascular conditions.

### Theme 2: Perceived effects of treatment

Seventeen (3%) within-trial responses at week 12 reported positive changes in signs or symptoms, such as, “very happy with blood pressure management” and “Less vague since starting the trial. Medication is excellent for me. Very happy.” Fourteen (4%) at week 52 had positive comments about signs or symptoms, “feels fantastic” and “Felt much better on this medication”. One respondent commented on feeling tired and lethargic at week 12, three (0.8%) had comments about negative effects at 52 weeks “anxiety mo(r)e obvious”, “light headedness…affected day-to-day life” and “experienced constipation and insomnia. felt initially had more energy but that then moved to insomnia which was disappointing”.

Of the 16 interviewees, 13 (81.3%) reported BP improvement during the QUARTET trial. The remaining three reported no change. Three participants stated they experienced adverse effects, including nausea, vomiting, diarrhoea, lethargy, presyncope, dry skin, and dry eyes. A separate three respondents reported experiencing a subjective reduction in heart rate, however, did not see this as an adverse effect. In comparison to their previous BP management, seven participants stated they experienced fewer adverse effects. Of the overall cohort, three reported a positive psychological change they associated with the study medication. For one person this stemmed from knowledge of the medication’s BP-lowering effectiveness: “I actually enjoyed having the low blood pressure and knowing that was actually good for me, and [that] motivated me” (interview 14). For the other two this stemmed from lifestyle improvement: “I went from not being able to get out of bed … to being able to get up and participate in life … it was just amazing” (interview 4), and “I felt healthier and happier and having a bit better lifestyle and didn’t feel so stressed all the time” (interview 6).

### Theme 3: Value of daily routine

All interviewees reported high adherence and stated they established a daily routine either prior to or during the QUARTET trial. Nine associated this routine with adherence during the trial. Participants reported convenience and adherence benefits in once-a-day dosing. Fourteen stated they took their medication in the morning, and some said this improved their adherence: “[For taking medication] the morning suits as that fits with my routine … if I took them at night I’d be less reliable, as the evenings are not so consistent” (interview 2). Breaks in normal routine was reported to increase the likelihood of forgetting to take medication, including forgetting in the evening or night due to tiredness or other commitments (*n* = 6), being in an unusual rush in the morning (*n* = 5), a family emergency (*n* = 2), COVID-19 pandemic (interview 13), and having a different routine on the weekend (*n* = 2) or on holiday (interview 2). Seven participants reported calendar packaging facilitated their adherence, as did being in a routine with their spouse (*n* = 2). Three participants stated symptoms would notify them if they were to hypothetically miss a dose. The remaining 13 stated as they would not experience symptoms if they hypothetically missed a dose, they would only realise whilst undertaking their daily routines.

### Theme 4: Individual responsibility

All interviewees stated daily adherence to medicines was solely their responsibility, but close family or friends were aware of their medications. Nine (56.3%) stored their medications in the kitchen, three the bedroom, three the bathroom, and one preferring an unspecified “cupboard away from the kids” (interview 5). The participants also described undertaking non-pharmacological methods they considered to improve BP control including stress reduction (*n* = 6), increased water consumption (*n* = 2), reduced salt intake or other dietary modifications (*n* = 6), weight loss (*n* = 3), increased exercise (*n* = 6), and meditation or other relaxation techniques (*n* = 2).

### Theme 5: Importance of the general practitioner

At week 12, three (1%) within-trial responses commented on following their GP’s advice for future treatment, e.g., “would not request specific medications but would be ruled by GP’s advice.” All interviewees recognised the role of their GP in their BP management. All reported they got their usual medications by obtaining a prescription from their GP and picking up the medication from their local pharmacist. Six paid a reduced amount due to government subsidies. Five highlighted the importance of their GP in not only their involvement in the QUARTET trial, but also in the adaptation of their BP management regimen to suit their individual preferences and responses to medication.

### Theme 6: Size and colour of capsule

Twenty-two (4%) within-trial respondents commented on the capsule being large and difficult to swallow at 12 weeks follow-up, and 12 (3%) made this comment at 12 months follow-up. Nine (2%) respondents commented on the capsule colour at the 12-week visit, with seven (78%) commenting the blue colour assisted identification of the study capsule. Four interviewees felt the size of the study’s capsule was large. However, these participants affirmed this did not deter or decrease their adherence, nor did it diminish convenience. As one interviewee said: “[the] size of the tablet was fine, my fish oil tablet is bigger than that” (interview 13). Conversely, one respondent said “the size of the pill was a little on the small side if I remember rightly” (interview 11), with another remarking the “tablets were the size of a (paracetamol) capsule” (interview 1).

### Theme 7: Experience of trial participation

Through the within-trial feedback some participants noted challenges with trial procedures and instructions; 8 (2%) at week 12 e.g., “work com(m)itments interfer(e)s with the trial visits” and “Have to have it with food so hard for a busy woman in the morning” and 6 (2%) at week 52, such as, “some difficulty in taking tablets on time due to extensive international travel” and “found it hard to take medication at regular time.” Other participants reported positive experiences of the study; 10 (2%) at week 12 e.g., “It has been very easy to be on this study” and 10 (3%) at week 52 “Study is well run. Good opportunity and beneficial to be a part of.”

All interviewees described having positive experience throughout their participation in the QUARTET trial. This primarily stemmed from the comprehensive evaluations: “everything was monitored to the 19th degree” (interview 10). Four respondents preferred the level of care within the trial compared to their experience with healthcare outside: “Medical care that I received within the trial was a lot more thorough than even going to the specialist” (interview 15). Further, integration of trial reports into their clinical care was seen positively (interview 1). Some reported increased knowledge regarding BP care, and both pharmacological and non-pharmacological methods of management: “[the QUARTET trial] highlighted a few things in my lifestyle that I needed to change that I wasn’t aware of, like drinking water” (interview 10). Aspects of interactions with trial staff appreciated by the participants (*n* = 6) included thorough explanation of BP management and trial procedures, good communication, and caring, empathetic and respectful attitude. BP monitoring for 24 h was the primary downside of the trial reported by seven interviewees, however all stated they understood it was necessary for testing the primary objective of the trial. Having to travel to or navigate unfamiliar locations during the trial was a point of inconvenience for three respondents. Without prompting, eight participants stated they hoped the study’s SPC would soon become commercially available.

## Discussion

This study adds to the literature the first characterisation of patient acceptability and attitudes towards the quadpill for blood-pressure lowering therapy. Our study identified six themes of patient acceptability of the quadpill and adherence: minimising medication, perceived effects, value of daily routine, individual responsibility, therapeutic relationship, and capsule characteristics. A seventh theme of experience of trial participation was also discussed. Through within-trial feedback from 85% of the trial participants we learned most found the study capsule to be easy or very easy to take, and most would request the quadpill if it was available through their usual doctor. Our participants valued once-a-day treatment with a single pill but would have preferred a smaller capsule. They understood their own role in adherence and the role of the GP in blood pressure management. SPCs may not be a one-size-fits-all solution, as individual patients may require different dose combinations, or experience different adverse effects. However, the findings of the present study suggest patients have positive attitudes, and significant acceptability, for quadpill therapy. This presents novel information potentially sufficient to modify existing clinical practice and public health policy when considered alongside the existing SPC evidence base, should the quadpill become available.

Observational, retrospective analyses of claims data suggest better adherence and outcomes with a single pill in comparison to multiple pills. Analysis of German statutory health fund claims demonstrated associations between standard-to-high dose SPCs including a renin-angiotensin system agent and lower mortality, reduced cardiovascular events, and greater persistence compared with multiple pills [23]. Healthcare utilisation data from Italy suggests those who receive a SPC of three drugs are more likely to have high adherence than those with a dual SPC plus another drug [24]. However these do not report the patients’ acceptability and views.

A study of a cardiovascular disease prevention polypill in primary healthcare found it generally acceptable, but more suitable for high-risk primary prevention [13]. A 2015 evaluation of the UMPIRE trial found most interviewees (trial participants and medical professionals) preferred the simplicity of a polypill, but had concerns it would be less tailored to individual needs [14]. A 2016 study found stroke survivors considered the concept acceptable, caregivers felt it would improve adherence, and GPs were open to prescribing it to those at increased risk of cardiovascular disease, however concerns were raised about lack of flexibility and suitability to the wider stroke population [15]. We see in the present evaluation participants find the concept of single-pill combination therapy acceptable in the context of initiating therapy or as an alternative to monotherapy. In their perspective there are benefits to the simplicity of low-dose single-pill once-a-day dosing fostering adherence.

Although blinded to study drug allocation, the QUARTET participants we interviewed expressed a positive view of the quadpill. As it is unlikely people would agree to participate in a drug trial if they had a negative view of the intervention, this was not surprising. But, as the average interval between completing the trial and being interviewed was about nine months we see this positive view has persisted. Comparing the within-trial feedback by treatment allocation showed some small (*n* = 5, 3%) differences at 12 months follow-up in the composite cells of “Average, difficult or very difficult” for ease of taking the trial medications, and “Unlikely or very unlikely” to request the quadpill if available from their usual doctor, but the vast majority of responses were highly positive for both groups at both time points so it is difficult to interpret this very small difference at 12 months. Interviewees recounted effective blood-pressure control with few adverse effects, with some reporting fewer compared to their usual medication.

Interviewees reported increased potential non-adherence stemmed from disruption to regular routines. Participants preferred morning for taking their medication and associated consumption in the evening with greater potential non-adherence, consistent with past literature investigating cardiovascular polypills [25]. Although it did not affect self-reported adherence in interviewees, and most feedback respondents reported it was easy to take the trial capsule, a small proportion found the capsule large. As larger pills are associated with increased rates of dysphagia and treatment failure [26], this is an important consideration for clinical implementation [7].

This study has several limitations. The number of interviews was determined by data saturation, and although this was reached with 16 participants, additional themes may have been elicited from a larger sample. Set questions within the interviews may have limited scope of responses, however these were open questions to promote freedom of discussion. Interviewees were aware of the study aims, and bias may have been introduced. To minimise this, an independent researcher conducted the interviews. Selection bias may be present, as participants who did not complete the QUARTET trial were excluded. Participants who agreed to participate in the interviews may have been more likely to report positive or extremely negative experiences. However, the quantitative analyses included all available data and captured comments on both positive and negative experiences.

## Conclusions

Trial participants reported positive views regarding the quadpill. Once-a-day morning dosing may benefit long-term adherence. Large capsule size did not impact self-reported adherence, but preference for smaller tablets was expressed. SPC blood-pressure lowering therapy appears acceptable in this population of people with high blood pressure previously untreated or on monotherapy. Together this evidence suggests the quadpill is acceptable to people with high blood pressure.

## Summary

### What is known about this topic

- Ultra-low dose quadruple combination therapy effectively lowers blood pressure
- There is general acceptability for single-pill combinations of three blood pressure drugs at standard doses

### What this study adds

- The majority of trial participants reported the capsule to be easy to take and they were likely to request it from their doctor if it was available
- Interviewees expressed preference for low-dose combinations, reduced pill burden, and once-a-day dosing

### Supplementary information

Supplementary appendix

## Data Availability

De-identified participant data that underlie the results reported in this article, will be available via application with a study proposal to the steering committee via the chair (clara.chow@sydney.edu.au). Data will only be shared with the approval of the steering committee and on signing of a data access agreement.
