# Supplementary material for: Participants’ views of ultra-low dose combination therapy for high blood pressure: a mixed-methods study from the QUARTET trial
Source: J Hum Hypertens. 2024 May 14;38(6):516–22. doi: 10.1038/s41371-024-00915-4 (PMC11166564; doi:10.1038/s41371-024-00915-4)
Supplement: Supplementary file 1 — Supplementary appendix [file 41371_2024_915_MOESM1_ESM.docx]

Supplementary appendix to

Participants’ views of ultra-low dose combination therapy for high blood pressure:

a mixed-methods study from the QUARTET trial

CORRESPONDENCE TO:

Dr Emily R. Atkins, The George Institute for Global Health, UNSW, Sydney, NSW 2000, Australia. Email: [eatkins@georgeinstitute.org.au](mailto:eatkins@georgeinstitute.org.au)

Contents

[Interview Guide 2](#_Toc164155034)

[Standards for Reporting Qualitative Research Checklist 4](#_Toc164155035)

[Supplementary Table 1 Response to feedback questions by age group, time since hypertension diagnosis, and polypharmacy at baseline 5](#_Toc164155036)

# Interview Guide

| **Your views and experience of the QUARTET trial** |
| --- |
| - Can you tell me a bit about how you came to be taking part in the QUARTET trial? - Can you tell me a bit about you experience on the trial? - Were there any aspects of the trial that were beneficial or problematic? - [If participant raises an area ] Ask how it compares to their experience of healthcare outside of the trial setting? - Now the study has finished do you feel there will be any changes for you? - Are there any ways the transition at the end of the trial back to usual care could be improved? If so, what would you have liked to have happened? - Was there anything about taking part in a trial that you were not expecting or that surprised you, either good or bad? - Have you taken part in research before? |
| **Your views and experience of LDQT and usual care** |
| - Can you tell me a bit more about your experience of taking the study drug during the trial? - What medications were you taking during the trial? - Can you tell me what you are taking that for? - If the participant says they take a medicine for X reason, is there any other ways you try to reduce your risk of X? Are there any ways you could reduce your risk of X that you find too difficult to do? - Were there any good points or bad points about the medicine you were taking during the trial? - During the trial have you experienced any side effects? |
| **Your views and experience of medications and your daily routine** |
| - How do you get hold of your supply of medications? - Do you have to pay for any of your medications? - Are there any other medicines or products for your health that you pay for? - Where do you normally keep your medication? - Can you tell me about your daily routine and the medications you take? |
| **Your views and experiences of taking your medications** |
| - So you have your tablets in front of you, can we go through them and talk a bit about each one? (e.g. colour, shape, packaging and size of pills, what they do) - In the last seven day can you describe if you’ve not taken any of these tablets and the reasons why? - In terms of taking you medications is there anyone who helps you to take your medications or do you help anyone else to take their medications? - Is anyone else aware of the medicines you are taking and do they know why you are taking them? - What factors or situations make it more or less likely to miss a dose? - How can you tell if you have missed a dose? - Are there some medications that you forget to take more often than others? |
| **Any other areas you would like to discuss?** |

# Standards for Reporting Qualitative Research Checklist

| Number | Topic | Location |
| --- | --- | --- |
| Title and abstract | | |
| S1 | Title | Title |
| S2 | Abstract | Abstract |
| Introduction | | |
| S3 | Problem formulation | Introduction, last paragraph |
| S4 | Purpose or research question | Introduction, last paragraph |
| Methods | | |
| S5 | Qualitative approach and research paradigm | Methods, paragraph 9 |
| S6 | Researcher characteristics and reflexivity | Methods, paragraph 3 |
| S7 | Context | Methods, paragraph 1 |
| S8 | Sampling strategy | Methods, paragraph 4 and paragraph 7 |
| S9 | Ethical issues pertaining to human subjects | Methods, paragraph 2 |
| S10 | Data collection methods | Methods, paragraph 4 and paragraph 7 |
| S11 | Data collection instruments and technologies | Methods, paragraph 4, paragraph 6, and Appendix I |
| S12 | Units of study | Methods, paragraph 4 and paragraph 7 |
| S13 | Data processing | Methods, paragraph 5 and paragraph 8 |
| S14 | Data analysis | Methods, paragraph 5 and paragraph 9 |
| S15 | Techniques to enhance trustworthiness | Methods, paragraph 9 |
| Results/findings | | |
| S16 | Synthesis and interpretation | Results paragraphs 5-13 |
| S17 | Links to empirical data | Results paragraphs 1-4, Tables 1, 2, 3 |
| Discussion | | |
| S18 | Integration with prior work, implications, transferability, and contribution to the field | Discussion, paragraphs 1-5 |
| S19 | Limitations | Discussion, last paragraph |
| Other | | |
| S20 | Conflicts of interest | Disclosures section, page 21 |
| S21 | Funding | Sources of funding, page 21 |

# Supplementary Table 1 Response to feedback questions by age group, time since hypertension diagnosis, and polypharmacy at baseline

|  |  | **During the trial, how easy did the participant find it to take the trial medications** | | | | **If the LDQT is available to be prescribed by participant’s usual doctor, how likely would the participant be to request it?** | | | | |
| --- | --- | --- | --- | --- | --- | --- | --- | --- | --- | --- |
|  |  | **Very Easy** | **Easy** | **Average, Difficult or Very Difficult** | **Total** | **Very likely** | **Likely** | **Average** | **Unlikely or Very Unlikely** | **Total** |
| **Week 12** |  |  |  |  |  |  |  |  |  |  |
| Age | <65 years | 251 (72%) | 81 (23%) | 15 (4%) | 347 | 206 (60%) | 100 (29%) | 28 (8%) | 12 (3%) | 346 |
|  | 65+ years | 119 (77%) | 30 (19%) | 6 (4%) | 155 | 81 (53%) | 55 (36%) | 12 (8%) | 6 (4%) | 154 |
| Time since hypertension diagnosis | <1 year | 176 (72%) | 59 (24%) | 11 (4%) | 246 | 131 (53%) | 84 (34%) | 24 (10%) | 6 (2%) | 245 |
|  | 1+ years | 194 (76%) | 52 (20%) | 10 (4%) | 256 | 156 (61%) | 71 (28%) | 16 (6%) | 12 (5%) | 255 |
| Medication count | <5 medications | 303 (73%) | 94 (23%) | 16 (4%) | 413 | 235 (57%) | 131 (32%) | 31 (8%) | 15 (4%) | 412 |
|  | 5+ medications | 67 (75%) | 17 (19%) | 5 (6%) | 89 | 52 (59%) | 24 (27%) | 9 (10%) | 3 (3%) | 88 |
|  |  |  |  |  |  |  |  |  |  |  |
| **Week 52** |  |  |  |  |  |  |  |  |  |  |
| Age | <65 years | 191 (74%) | 52 (20%) | 14 (5%) | 257 | 166 (65%) | 73 (28%) | 12 (5%) | 6 (2%) | 257 |
|  | 65+ years | 84 (82%) | 15 (15%) | 3 (3%) | 102 | 44 (44%) | 39 (39%) | 11 (11%) | 7 (7%) | 101 |
| Time since hypertension diagnosis | <1 year | 127 (77%) | 33 (20%) | 5 (3%) | 165 | 86 (52%) | 59 (36%) | 13 (8%) | 6 (4%) | 164 |
|  | 1+ years | 148 (76%) | 34 (18%) | 12 (6%) | 194 | 124 (64%) | 53 (27%) | 10 (5%) | 7 (4%) | 194 |
| Medication count | <5 medications | 224 (76%) | 57 (19%) | 13 (4%) | 294 | 168 (57%) | 98 (33%) | 17 (6%) | 10 (3%) | 293 |
|  | 5+ medications | 51 (78%) | 10 (15%) | 4 (6%) | 65 | 42 (65%) | 14 (22%) | 6 (9%) | 3 (5%) | 65 |
